# Direct Esthetic Rehabilitation of Teeth with Severe Fluorosis: A Case Report

**Published:** 2014-03

**Authors:** F. Shafiei, MS. Tavangar, AA. Alavi

**Affiliations:** a Biomaterial Research Center, Dept. of Operative Dentistry, School of Dentistry, Shiraz University of Medical Sciences, Shiraz, Iran

**Keywords:** Fluorosis, Esthetic, Direct composite veneer, Fiber- reinforced composite, bridge

## Abstract

This article describes an esthetic rehabilitation of a case of severe fluorosis associated with tooth mobility and gingival recession. Direct composite technique was applied to improve the color, shape and alignment of the teeth using direct composite veneering and replacement of the missing tooth by fiber-reinforced composite bridge. One year follow up have displayed acceptable outcomes and esthetic appearance.

## Introduction

Dental fluorosis is caused by an excessive fluoride intake during tooth formation. Fluoride-containing dental products and drinking water are two main potential sources for this developmental tooth disorder. Fluoride-related alterations in enamel lead to surface hypermineralization and subsurface hypomineralization which are characterized by white opaque appearance with secondary brown stain [1-2].

The successful treatment of fluorosed teeth is a subject of interest in the literature. An appropriate treatment plan may be selected depending on the severity of the fluorosis [1-2]. In the mild cases of dental fluorosis, clinical appearance is characterized by opaque white areas presenting as horizontal lines and cloudy patches on the enamel surface. Bleaching and microabrasion have been recommended for these forms of fluorosis. In the moderate-to-severe level of fluorosis, all tooth surfaces are affected by white opacities [1-2]. Brown stains also present in the involved teeth. Some pits and wear area may be observed on the surfaces as a result of damage to the poorly mineralized enamel. Treatments include microabrasion, direct composite restorations or combination of both methods. In some instances, esthetic veneers or crowns may be necessary for the some patients [1-2].

Frequently, the management of fluorosis involves resin composite restorations. In this situation, some concerns raise about the effect of etching and bonding agents on the fluorotic enamel and dentin [2]. This article presents the stages of esthetic rehabilitation of a patient with severe fluorosis including direct composite veneering and fiber-reinforced composite bridge.

## Case Presentation

**Figure 1:**
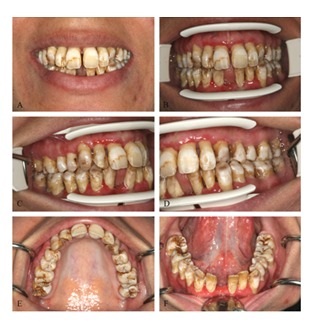
Preoperative clinical pictures: **A** Smile.  **B** Frontal view.  **C** Lateral view, right side.  **D** Lateral view, left side.  E Maxillary arch, occlusal view.  F Mandibular arch, occlusal view.

A 21-year-old woman was referred to the department of operative dentistry in the faculty of dentistry for the esthetic rehabilitation. The patient was not satisfied with her smile appearance because of the discolored tooth and excessive length of maxillary incisors. She also needed replacement of the extracted teeth (Figure 1).

**Figure 2:**
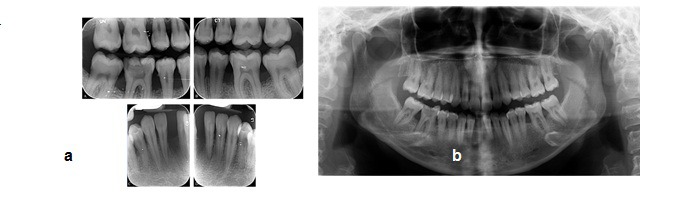
**a** Preoperative Panoramic radiograph  **b** Preoperative intraoral radiographs

She was healthy, having no systemic diseases and she did not have any contraindication for dental procedures.

The patient’s dental history showed an aggressive periodontitis in her adolescence. Radiographic evaluation and clinical examination revealed bone loss especially in the area of mandibular incisors and first molars which led to mobility of the other incisors (Figure 2). Oral hygiene was assessed to be good with the gingival and plaque index of below 10%.

**Figure 3:**
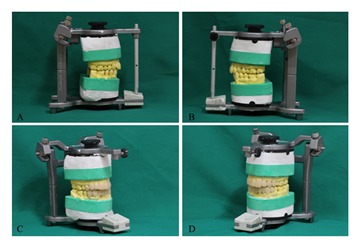
**A** and **B** Preoperative maxillary and mandibular full arch records.  **C** and **D** diagnostic wax up

The patient presented generalized areas of fluorosis representing as opaque patches, subsurface brown staining and small pits in enamel representing severe fluorosis. The diagnosis was made based on the Dean's fluorosis index. The clinical and diagnostic cast examinations revealed the rotation of tooth #7, the cross bite in tooth#11, excessive length of clinical crowns of maxillary central teeth and flaring of the anterior teeth proceeding to upper midline diastema (Figure 1). At the time of evaluation, no restoration of the teeth was present. No active caries was detected except for the teeth #19 and #30. The root canal therapies of tooth #30 and the amalgam restoration of teeth #19 and #30 were performed. The maxillary and mandibular full arch impressions and inter-occlusal records were performed. A diagnostic wax-up was made using direct restorative procedure (Figure 3).

The color was recorded using the Vita Classical shade guide, and the shade A2 and A3 was considered as the initial color.

The tooth preparation involved a minimal chamfer in the facial surfaces and additional reduction for correcting the length and orientation of teeth was limited to these areas. Cotton rolls, salivary ejectors, and retraction cords were used for field isolation. The enamel surface was acid etched using 35% phosphoric acid gel (Ultra- Etch; Ultradent, South Jordan, UT, USA) for 15 second, rinsed for 10 seconds and dried. A self-etch, two-component adhesive system (Clearfil SE bond; Kuraray, Osaka, Japan) was applied on the prepared enamel and dentin surface and light-cured for 10 second with an intensity of 1100 mW/cm2 (Demi Plus LED; Kerr, Middleton, USA). Direct composite veneers were contoured regarding the diagnostic wax up. A stratified layering technique was used to fill the tooth with microhybrid resin composite (Vit-l-escence;Ultradent, South Jordan, UT, USA) shade A2, 3 and Pearl Neutral. The diastema was closed and the alignment and length of teeth were corrected where it was necessary. The composite was light-cured through the matrix for 10 seconds on each surface. After removal of the matrix, each surface was polymerized from incisal/occlusal, facial, and lingual aspects for additional 20 seconds. 

**Figure 4:**
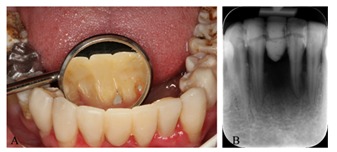
**A** Fiber-reinforced composite bridge. **B** splint design of mandibular incisors.

The fiber-reinforced composite bridge was made in order to replace tooth #25 and splint the mobile mandibular incisors simultaneously. An appropriate length of polyethylene fiber (Fiber-Braid;NSI, Dental Pty Ltd, New South Wells, Australia) was cut and saturated with bonding agent (Margin Bond; Coltène-Whaledent, Altstätten, Switzerland).The interproximal and lingual surfaces were prepared and treated as described before. Flowable composite (PermaFlo; Ultradent, South Jordan, UT, USA) was used to make interproximal composite connectors and cover the splinting fiber (Figure 4).

Finally, the occlusal adjustment of the restorations was performed and the canine guidance discluded all the posterior teeth in the eccentric mandibular movements.

The contouring and finishing was accomplished with finishing burs (Composite Finishing Bur Kit; Ultradent, South Jordan, UT, USA). The polishing was performed using polishing disk, polishing points and cups (Jiffy Polishers and Jiffy HiShine; Ultradent, South Jordan, UT, USA) and diamond polishing paste (Diamond Polish Mint; Ultradent, South Jordan, UT, USA).

**Figure 5:**
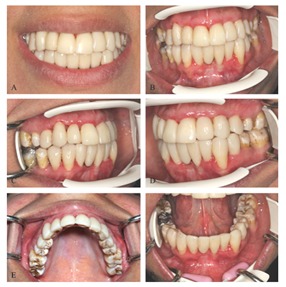
Postoperative clinical pictures:  **A** Smile. **B** Frontal view.  **C** Lateral view, right side.  **D** Lateral view, left side.  **E** Maxillary arch, occlusal view.  **F** Mandibular arch, occlusal view.

The smile of the patient revealed good esthetics after the performed treatments (Figure 5). Adequate oral home-care regimen was strongly emphasized for the patient. The patient was visited every 3 months for monitoring of the restorations placed.One-year follow up showed good aspect without any debonding, secondary caries, pain and sensitivity.

## Discussion

The aim of the treatment in this case was to restore the patient esthetics and self-esteem. Different treatment plans have been proposed for the treatment of discoloration in the fluorosed teeth depending on the severity of the fluorosis [2]. Since some alteration in the shape and the contour of teeth was required, satisfactory results could not be achieved by the microabrasion technique. Another factor was the periodontal support of the teeth; regarding the mobility and questionable prognosis of some teeth, coverage of the teeth with porcelain veneers or crowns was not feasible. A direct composite restoration was a conservative alternative which offered the ability to correct the shape and the contour of teeth in addition to the removal of discoloration. Furthermore, the replacement of the extracted tooth was possible by using direct composite in making fiber-reinforced composite bridge. Direct resin bonded bridge is a conservative choice for replacing one, or several teeth, employing reinforced- fiber materials. If the missing teeth are associated with periodontal problem, fiber- reinforced composite (FRC) bridge which includes a splint design is indicated [3].

Although using direct composite provides excellent esthetics; the fracture resistance, wear resistance and color stability of composite resin is lower than indirect porcelain restorations [4]. Furthermore, bonding procedure to the fluorosed enamel and dentin can be challenging. However, reliable bond strength to the fluorosed enamel has been reported in the mild or moderate cases. In these cases some modifications in the preparation etching time and selection of adhesive system had been done [5-6].

It is recommended to grind the fluorosed enamel surface to remove the hypermineralized layer. Etching with phosphoric acid for 15 seconds achieved the best results in the normal enamel [7] . While the best etching result were obtained at 30 seconds for the moderate fluorosed enamel, increased etching time for severe fluorosis result in less retentive surface [7] . The bond strength of all the adhesive systems to enamel is adversely affected by fluorosis. Etch-and-rinse systems provide the highest bond strength to fluorosed enamel [8]. Separate steps of etching and rinsing are required with self-etch adhesive in the case of moderate and severe enamel fluorosis [8]. In contrast to surface enamel, fluorosed dentin is more susceptible to acid, especially in the severely affected teeth. Therefore, etch-and-rinse systems are not recommended for bonding the dentin in the affected teeth [9-10]. It has been reported that reliable adhesion can be obtained using two-step self-etch adhesive system [9-10].
